# Transcriptional profiling of the murine cutaneous response during initial and subsequent infestations with *Ixodes scapularis *nymphs

**DOI:** 10.1186/1756-3305-5-26

**Published:** 2012-02-06

**Authors:** Dar M Heinze, Stephen K Wikel, Saravanan Thangamani, Francisco J Alarcon-Chaidez

**Affiliations:** 1 Department of Pathology, University of Texas Medical Branch, Galveston, Texas 77555, USA; 2 Department of Basic Medical Sciences, Quinnipiac University School of Medicine, Hamden, CT 06518, USA; 3 Institute for Human Infections and Immunity, University of Texas Medical Branch, Galveston, Texas 77555, USA

**Keywords:** Tick(s), *Ixodes scapularis*, Skin, Cutaneous response, Tick-host interface, Gene expression profiling

## Abstract

**Background:**

*Ixodes scapularis *ticks are hematophagous arthropods capable of transmitting many infectious agents to humans. The process of blood feeding is an extended and continuous interplay between tick and host responses. While this process has been studied extensively *in vitro*, no global understanding of the host response to ticks has emerged.

**Methods:**

To address this issue, we used PCR-arrays to measure skin-specific expression of 233 discrete genes at 8 time points during primary and secondary infestations of mice with pathogen-free *I. scapularis *nymphs. Selected results were then validated at the mRNA and protein levels by additional real-time PCR and bioplex assay.

**Results:**

Primary infestation was characterized by the late induction of an innate immune response. Lectin pattern recognition receptors, cytokines, and chemokines were upregulated consistent with increased neutrophil and macrophage migration. Gene ontology and pathway analyses of downregulated genes suggested inhibition of gene transcription and Th17 immunity. During the secondary infestation, additional genes were modulated suggesting a broader involvement of immune cells including CD8 and CD4 positive T lymphocytes. The cytokine response showed a mixed Th1/Th2 profile with a potential for T regulatory cell activity. Key gene ontology clusters observed during the secondary infestation were cell migration and activation. Matrix metalloproteinases were upregulated, apoptosis-related genes were differentially modulated, and immunoreceptor signaling molecules were upregulated. In contrast, transcripts related to mitogenic, WNT, Hedgehog, and stress pathways were downregulated.

**Conclusions:**

Our results support a model of tick feeding where lectin pattern recognition receptors orchestrate an innate inflammatory response during primary infestation that primes a mixed Th1/Th2 response upon secondary exposure. Tick feeding inhibits gene transcription and Th17 immunity. Salivary molecules may also inhibit upregulation of mitogenic, WNT, Hedgehog, and stress pathways and enhance the activity of T regulatory cells, production of IL-10, and suppressors of cytokine signaling molecules (SOCS). This study provides the first comprehensive transcriptional analysis of the murine host response at the *I. scapularis *bite site and suggests both a potential model of the host cutaneous response and candidate genes for further description and investigation.

## Background

The process of tick feeding activates a highly complex sequence of events at the bite site that facilitate the acquisition of a blood meal and create a suitable microenvironment for pathogen transmission and establishment [1]. These events are governed by an array of salivary molecules secreted by the tick and the responses of the host to those molecules. It is a dynamic relationship with outcomes ranging from successful tick engorgement and potential pathogen transmission to tick rejection and greatly reduced pathogen acquisition. A critical factor that controls this variability is the host response to tick feeding. Laboratory animals with prior exposure to ticks may be significantly protected from pathogen acquisition from infected ticks [2]; after a single feeding with *Dermacentor variabilis*, rabbits develop an anti-tick immunity that greatly reduces successful blood feeding during future infestations [3]. These observations suggest the host response to infestation may yield vital insights for tick and tick-borne disease control.

During the course of blood feeding, ticks have been shown to inhibit host pain/itch responses, hemostasis, angiogenesis, complement activation, and both innate and adaptive immune responses. *In vitro *experiments suggest tick saliva inhibits the production of cytokines (IL-1, IL-2, IL-6, IL-12, TNF-α, and IFN-γ) and adhesion molecules (ICAM-1, VCAM-1, P-selectin, LFA-1, and VLA-4) with the notable exception of IL-4 and IL-10 [1]. The production of IL-4 in response to tick feeding has been supported *in vivo *[4]. Tick salivary molecules also inhibit the function of immune cells present at the bite site. Salp15, an *I. scapularis *salivary protein, inhibits CD4-mediated activation of helper T-cells [5] and modulates dendritic cell activation through the lectin receptor DC-SIGN [6]. Similarly, salivary gland disintegrin-like proteins ISL 929 and ISL 1373 inhibit neutrophil function [7] while salivary gland extracts have been shown to inhibit dendritic cell maturation, migration, and cutaneous turnover [8]. Despite the ability of tick saliva to suppress host responses, some animals develop successful immunity dependent in part on T-cells, antibodies, complement, mast cells, and basophils [9]. Piper and colleagues have compared the gene expression profile in skin [10,11] and white blood cells [12] of tick resistant *Bos indicus *and tick susceptible *Bos taurus *cattle after multiple artificial and natural infestations with *Rhipicephalus microplus*. These studies suggest T-cell mediated immunity, integrity of the dermis, and calcium signaling are important aspects of tick resistance, while innate immune responses may contribute to susceptibility. Thus our present understanding indicates host immunity to ticks is characterized by a complex interplay between host-effector responses and tick evasion strategies.

The tick-host interface is the skin, an organ increasingly recognized to have a significant role in immunity, acting as a sentinel organ that also shapes the ensuing immune response [13]. Anatomically, the skin is divided into two compartments, the epidermis and dermis. The barrier function of the epidermis is maintained by keratinocytes, while keratinocytes, lymphocytes, and langerhans cells play a role responding to epidermal invasion [13]. The dermal compartment is much more heterogeneous, with lymphocytes, macrophages, mast cells, natural killer cells, fibroblasts, and multiple types of dendritic cells [13]. In addition, lymphatic and vascular channels allow the migration of many additional cell types into the dermis. Thus the skin presents a complex array of resident and circulating cells that participate in homeostasis, immunosurveillance, and immune responses. In the case of tick feeding, the cutaneous response represents both the initiation and effector functions of the host. In an effort to understand the spectrum and temporal patterns of the *in vivo *host response to ticks, we used a PCR-array based approach to characterize the patterns of cutaneous bite-site gene expression during the course of primary and secondary infestations of mice with *I. scapularis *nymphs.

## Methods

### Ticks

Pathogen-free *I. scapularis *colonies were maintained in our laboratory as described [14]. All life cycle stages were kept in sterile glass vials with mesh tops in desiccators at 22°C containing saturated salt solutions to obtain 97% relative humidity with a 16:8 hour photoperiod. For routine colony maintenance adult ticks were fed on New Zealand white rabbits and nymphs and larvae were fed on mice.

### Time course infestations

To perform time course infestations, six-week-old female BALB/c mice were placed in individual restrainers and infested with 10-15 pathogen-free *I. scapularis *nymphs. Ticks were allowed to attach for at least one hour and unattached ticks were discarded. Mice were then removed from restraints and housed individually. Secondary infestations involved two rounds of infestation. Mice were infested with 10-15 *I. scapularis *nymphs that were allowed to complete their feeding cycle (4-5 days). Fourteen days after the last primary infestation tick dropped off the animals, mice were re-infested with 10-15 *I. scapularis *nymphs. For tissue harvesting, infested mice were euthanized by CO_2 _inhalation followed by cervical dislocation and 4 mm punch biopsies were taken from the feeding lesion at 12, 48, 72, and 96 hr post-infestation. Three mice were measured at each time point; controls consisted of 3 similarly housed but tick-free mice. Biopsies were stored in RNAlater (Ambion) at -20°C for RNA and snap frozen in liquid nitrogen and stored at -80°C for cytokine analysis. The Institutional Animal Care and Use Committee of the University of Texas Medical Branch approved all animal experiments (protocol #0907054).

### RNA Isolation

Mouse tissues stored in RNAlater (Ambion) were used for RNA extraction by a combination of Trizol reagent (Invitrogen) and RNeasy (Qiagen) protocols that included an in-column DNase digestion step. Quality and integrity of RNA was verified by the ratio of readings at A_260_/A_280 _and A_260_/A_230 _(all samples read ≥ 1.8 by both ratios), and by denaturing (formaldehyde) agarose gel electrophoresis followed by staining with Sybr Gold stain (Invitrogen). All samples had readily visible 18S and 20S RNA bands, indicating minimal degradation. Eluted RNA samples were aliquoted and stored at -80°C until use.

### Host gene expression profiling using pathway-specific PCR Array analysis

Host cutaneous gene expression was assessed at each time point using three commercially available RT^2 ^Profiler PCR Arrays (SAbiosciences, now Qiagen). Arrays were chosen to measure biological pathways related to T-helper cell differentiation (PAMM-073), wound healing (PAMM-013), and signal transduction (PAMM-014). Each 96-well array contains 84 test and five housekeeping genes. Each array also included controls to assess genomic DNA contamination, RNA quality, and general qRT-PCR performance (for more information, see http://www.sabiosciences.com/). For each array, 1 μg total RNA purified from skin biopsies was converted into cDNA using the RT^2 ^First strand kit (Qiagen). Template cDNAs were mixed with RT^2 ^SYBR Green/Fluorescein qPCR Master Mix (Qiagen) and loaded onto the array using an 8-channel pipette. Arrays were run on an iCycler iQ5 real-time PCR System (Bio-Rad) under standard cycling conditions (10 min at 95°C, 15 s at 95°C, 1 min 60°C for 40 cycles, and an 80-cycle 55-95°C melt curve). The instrument's software was used to calculate the threshold cycle (*C_t_*) values for all molecules analyzed.

### Array data analysis

Fold-changes in gene expression between test and control mice were calculated using the ΔΔCt method. For each included gene, individual measurements that were below the threshold selected (*C_t _*> 34) were excluded from further analysis. This was done to reduce the impact of stochastic variations in rare transcripts on the calculated fold change and its associated p-value. Data normalization was based on correcting all *C_t _*values for the average *C_t _*values of the invariant endogenous control genes hypoxanthine guanine phosphoribosyl transferase (Hprt) and heat shock protein 90 alpha (Hsp90ab1). These genes were selected based on analysis of tested housekeeping genes in geNorm [15]. Statistical significance was assessed using LIMMA (linear models in micro-array analysis) in HTqPCR, an R-based program designed for real-time PCR array data analysis [16]. Statistical comparisons were generated for all time points vs. uninfested controls, between time-points (i.e., 12 hr-48 hr, 48 hr-72 hr, etc.), and between infestations (e.g. 12 hr primary vs. 12 hr secondary, etc.). Data sets were filtered with the following criteria: fold change ≥ 3 or ≤ -2 with an adjusted p-value ≤ 0.01. Array data was made publicly available through Gene Expression Omnibus accession number GSE33345.

### Gene ontology

Gene ontology analysis was conducted on the resulting lists of significantly modulated genes. All significant results from any time point during the primary infestation were divided into three lists: all modulated, upregulated, and downregulated. Similar lists were created for the secondary infestation. Each list was then submitted to the Database for Annotation, Visualization, and Integrated Discovery (DAVID) website using all genes measured as a background list [17,18]. The functional annotation chart and functional annotation clustering tools were used to assess enriched gene ontology terms; due to the small background list, terms with p-values ≤ 0.05 were considered significant.

### Validation of array data

Array results were validated by an additional experiment. Skin biopsies from tick bite-sites were collected as before from two time points during primary (48 and 96 hr p.i.) and secondary (48 and 72 hr p.i.) infestation. Four mice were used at each time point. Twenty-five genes were selected from the list of significantly modulated genes from the array experiment and assayed by additional real-time PCR (see additional file 1). Primer assays and SYBR green master mix were purchased from Qiagen and added to PCR plates to make custom-made arrays. These primer assays contain pre-optimized primer pairs but the primer sequences are proprietary information of Qiagen. Custom-made arrays measured the same 5 house-keeping genes as the original arrays, and included both no template and no first strand controls. In contrast to the arrays, each gene was measured in triplicate. These plates were run and analyzed as the PCR arrays, including the melt curve. To keep data analysis consistent with the PCR arrays, Hprt and Hsp90ab1 were used as normalization genes without additional analysis with geNorm.

### Cytokine analysis

The relative concentrations of interleukin-1β (IL-1β), IL-3, IL-4, IL-6, IL-10, IL-17A, interferon-γ (IFN-γ), and monocyte chemoattractant protein-1 (MCP-1 or CCL2) at the tick bite site were quantified using an 8-analyte bioplex assay and the Bioplex 200 system (Bio-Rad). Samples represented two time points (4 mice/time point) in the primary and secondary infestations (48 hr and 96 hr primary, and 48 hr and 72 hr secondary). Biopsies were removed from storage at -80°C and immediately homogenized in 1 mL protein extraction buffer containing 0.5% BSA (Fisher Scientific), 0.1% Igepal-630 (Sigma), and 1% Halt protease inhibitor (Thermo Scientific) in PBS. Homogenates were centrifuged at 20,000 × g at 4°C for 20 min. The resulting supernatants were divided into aliquots and stored at -80°C until use. Standards, blanks, and samples were analyzed in duplicate according to the manufactures' instructions. Analyte concentrations were determined from the standard curve(s) by analysis of mean fluorescent intensity values using the Bio-Plex Manager™ 6.0 software. Individual time points were compared to controls using a 2-tailed T-test in Prism (GraphPad Software, Inc.).

## Results

**Figure 1:**
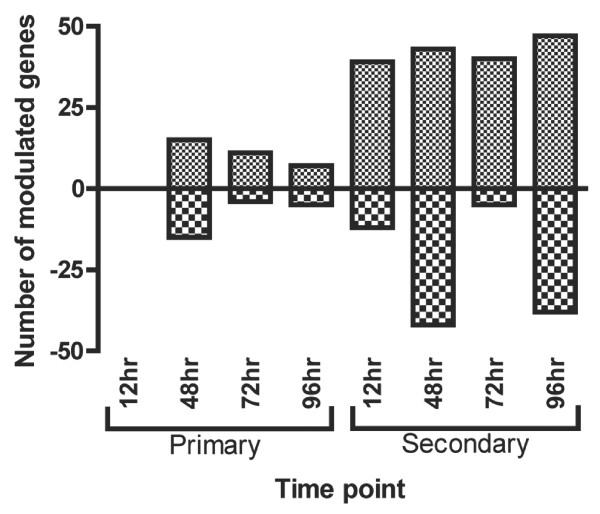
**Number of modulated genes at each time point compared to tick-free mice**. Number of significantly up and downregulated genes measured at each time point during primary and secondary infestations of mice with *I. scapularis *nymphs compared to tick-free mice.

In this study, Balb/cJ mice were infested with nymphal *I. scapularis *ticks and the expression of 233 gene transcripts were measured at the bite site lesion during primary and secondary infestations. These results revealed a distinct expression profile in naïve mice that was markedly different from that observed following a secondary infestation. Based on the selection criteria for differentially regulated genes (Materials and Methods), we identified 35 genes that differed in expression during primary infestation (17 upregulated and 18 downregulated) and 138 genes that differed during secondary infestation compared to uninfested control mice. The total numbers of differentially expressed genes when compared to control mice are illustrated in Figure 1. Fold changes ranged from negative 24-fold (LEP, secondary infestation at 12 hr post-infestation (p.i.)) to over 3000-fold (CXCL5, secondary infestation at 96 hr p.i.). Results from the primary infestation did not show any significant changes in gene expression at 12 hr p.i. when compared to control mice. At 48 hr p.i., however, significant modulation of gene expression was observed which gradually reduced towards the end of the feeding period (96 hr). As expected, many additional genes were modulated during secondary infestation. Overall, numbers of upregulated genes remained fairly stable across different time points as well as within each infestation scheme while a more variable response was observed for downregulated or unresponsive genes (Figure 1). Statistical evaluation using linear models in microarray analysis (LIMMA) did not show any significant changes in expression between time points within an infestation scheme; however, significant results were obtained when comparing expression levels between primary and secondary infestations (additional file 2).

Gene ontology (GO) analysis was undertaken to assess potential biological functions represented in our gene lists (Tables 1, 2, and 3). GO-terms enriched from genes upregulated during the primary infestation (Table 1) clustered into two categories: host response and biomineral formation. In the host response category, the majority of GO-terms were related to chemotaxis, cytokines, immune response, and cellular location while a single term was observed in the category of biomineral formation. This category deals with all aspects of development of hard tissues that consist mainly of inorganic compounds and small amounts of organic matrices. Genes that were downregulated during the primary infestation were enriched for GO-terms that fell into two clusters: nucleotide metabolism/transcription and Similar Expression to Fibroblast Growth Factor and IL-17Rs or SEFIR (an IL-17 receptor domain) (Table 2). The former category contained terms related to gene expression and metabolic processes while the SEFIR category contains domains known to participate in IL-17RA signaling independently of classic Toll/IL-1R (TIR) structures including MyD88 and TRIF [19].

**Table 1:** Gene ontology results from genes upregulated during primary infestation.

Cluster	Term	PValue
Host response	Chemokine activity	0.005
	Chemokine receptor binding	0.005
	Chemokine signaling pathway	0.014
	Small chemokine, interleukin-8-like	0.0031
	CXC chemokine	0.047
	Cytokine	0.0009
	Cytokine activity	0.0041
	Cytokine-cytokine receptor interaction	0.015
	NOD-like receptor signaling pathway	0.001
	Extracellular region part	0.025
	Extracellular space	0.0036
	Secreted	0.0097
	Immune response	0.008
	Inflammatory response	0.0012
	Defense response	0.0003
	Response to wounding	0.0039
	Taxis	0.0007
	Chemotaxis	0.0007
	Locomotory behavior	0.0007
	Behavior	0.006

Biomineral formation	Biomineral formation	0.024

Significant gene ontology terms from genes upregulated at any time point during primary infestation of mice with *I. scapularis *nymphs are shown grouped into clusters based on the functional annotation clustering tool available from DAVID. Terms in the host response cluster have been grouped by similarity.

**Table 2:** Gene ontology results from genes downregulated during primary infestation.

Cluster	Term	PValue
Nucleotide metabolism	Cell morphogenesis	0.039
and transcription	DNA binding	0.020
	Positive regulation of biosynthetic process	0.025
	Positive regulation of cellular biosynthetic process	0.022
	Positive regulation of gene expression	0.046
	Positive regulation of macromolecule biosynthetic process	0.022
	Positive regulation of macromolecule metabolic process	0.042
	Positive regulation of nitrogen compound metabolic process	0.014
	Positive regulation of nucleobase, nucleoside, nucleotide and nucleic- acid metabolic process	0.010
	Positive regulation of RNA metabolic process	0.032
	Positive regulation of transcription	0.036
	Positive regulation of transcription from RNA polymerase II- promoter	0.025
	Positive regulation of transcription, DNA-dependent	0.032
	Regulation of RNA metabolic process	0.025
	Regulation of transcription	0.046
	Regulation of transcription from RNA polymerase II promoter	0.027
	Regulation of transcription, DNA-dependent	0.027
	Sequence-specific DNA binding	0.018
	Tissue morphogenesis	0.026

SEFIR domain	IPR013568:SEFIR (IL-17R domain)	0.026

Significant gene ontology terms from genes downregulated at any time point during primary infestation of mice with *I. scapularis *nymphs are shown grouped into clusters based on the functional annotation clustering tool available from DAVID.

**Table 3:** Gene ontology results from genes upregulated during secondary infestation.

Cluster	Terms	PValue	Cluster	Terms	PValue
**Putative**	Disulfide bond	0.00002	**Immune cell**	Positive regulation of signal transduction	0.0032
**Secreted**	Signal peptide	0.0038	**signaling and**	Positive regulation of cell communication	0.005
	Signal	0.0043	**activation**	Four-helical cytokine, core	0.0061
	Glycoprotein	0.013		Regulation of cell activation	0.0063
**Cytokine**	Immune response	0.00001		Regulation of peptidyl-tyrosine phosphorylation	0.0071
			
	Cytokine	0.00026		Regulation of leukocyte proliferation	0.008
	Cytokine activity	0.0013		Regulation of T cell activation	0.008
	Extracellular space	0.0019		Positive regulation of peptidyl-tyrosine phos.	0.011
	Cytokine-cytokine receptor interaction	0.0054		Regulation of lymphocyte activation	0.014
			
**Hematopoietic**	Hematopoietic cell lineage	0.0011		Regulation of leukocyte activation	0.014
**lineage**	T-cell	0.0032		Regulation of hematopoiesis by cytokines	0.019
	Regulation of hematopoiesis by cytokines	0.019		Regulation of mononuclear cell proliferation	0.019
			
**Inflammation**	Inflammatory response	0.027		Regulation of lymphocyte proliferation	0.019
	Defense response	0.027		Regulation of protein kinase cascade	0.02
			
**Chemotaxis**	Chemotaxis	0.0024		Positive regulation of T cell activation	0.03
	Locomotory behavior	0.0024		Regulation of cellular localization	0.033
	Taxis	0.0024		Positive regulation of protein kinase cascade	0.033
	Leukocyte adhesion	0.017		Regulation of protein modification process	0.034
	Cell chemotaxis	0.026		Regulation of cellular protein metabolic process	0.034
	Leukocyte chemotaxis	0.026		Positive regulation of immune system process	0.034
	Neutrophil chemotaxis	0.026		Regulation of chemokine production	0.039
	Leukocyte migration	0.032		Regulation of chemokine biosynthetic process	0.039
			
**Cell surface**	Monocyte and its Surface Molecules	0.0025		Positive regulation of cell activation	0.045
**molecules**	Cell adhesion molecules (CAMs)	0.0071		Positive regulation of protein modification process	0.047
	External side of plasma membrane	0.01		Regulation of T cell proliferation	0.047
			
	Cell surface	0.021	**Tyrosine**	Regulation of peptidyl-tyrosine phosphorylation	0.0071
			
**T-cell regulation**	Positive regulation of T cell activation	0.03	**phosphorylation**	Positive regulation of peptidyl-tyrosine phos.	0.011
	Regulation of cellular localization	0.033	**Leukocyte**	Monocyte and its Surface Molecules	0.0025
			
	Regulation of alpha-beta T cell differentiation	0.039	**adhesion**	Cell adhesion molecules (CAMs)	0.0071
	Positive regulation of alpha-beta T cell diff.	0.039		Neutrophil and Its Surface Molecules	0.0082
			
**Sushi domain**	domain:Sushi 2	0.036		Leukocyte adhesion	0.017
	domain:Sushi 1	0.036		Adhesion Molecules on Lymphocyte	0.019
	domain:EGF-like	0.036		Natural killer cell mediated cytotoxicity	0.021
			
**Activation**	Propeptide:Activation peptide	0.048			
**peptide**					

Significant gene ontology terms from genes upregulated at any time point during secondary infestation of mice with *I. scapularis *nymphs are shown grouped into clusters based on the functional annotation clustering tool available from DAVID.

In contrast with the primary infestation, upregulated transcripts during secondary exposure were enriched for GO-terms related to a wide array of categories as shown in Table 3. From this data, four major categories are evident: cytokine, chemotaxis, immune cell signaling and activation, and leukocyte adhesion. Other groups of enriched terms included T-cell regulation and cell surface molecules while the remaining terms clustered into a number of minor categories related to putative secreted, hematopoietic lineage, inflammation, protein-protein interactions (sushi domain), activation peptide, and tyrosine kinase phosphorylation. On the other hand, only GO terms 'negative regulation of cell proliferation' (GO_BP:0008285) and SEFIR (IPR013568:SEFIR) were significantly enriched from genes downregulated in the secondary exposure.

### Modulation of gene expression during primary infestations

**Figure 2:**
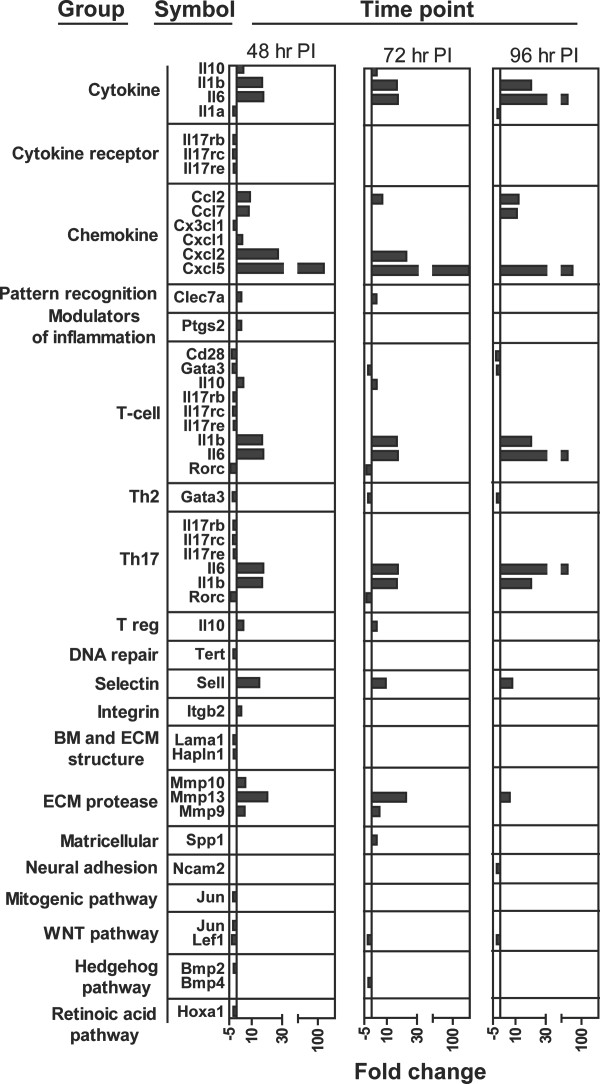
**Genes modulated during primary infestations of mice compared to tick-free mice**. Gene expression was measured using PCR arrays at 12, 48, 72, and 96 hr p.i. during a primary exposure of BALB/cJ mice to *I. scapularis *nymphs. Significantly modulated genes were divided into biologically meaningful groups (Methods) to allow direct comparison between time points. Official gene symbols and fold changes for all results at 48, 72, and 96 hr p.i. are shown. No significant gene modulation was apparent at 12 hr p.i.

While gene ontology allows assessment of inapparent biological processes in a list of genes, it does not allow direct comparison between time points or infestations at the gene level. To facilitate this, all 233 genes measured were divided into individual groups based on shared characteristics of the translated protein (shared family, pathway, or function). These groups and the genes in each group can be accessed in additional file 3. Genes modulated during the primary infestation are shown in Figure 2. Upregulated genes that were consistently expressed during the course of tick feeding included cytokines IL-10, IL-6, and IL-1β, chemokines CCL2, 7, CXCL1, 2, and 5, pattern recognition receptor CLEC7a, modulator of inflammation prostaglandin-endoperoxide synthase 2 (PTGS2 or COX2), extracellular matrix (ECM) proteases MMP9, 10, and 13, and the adhesion molecules L selectin (SELL), and β-2 integrin (ITGB2).

Among downregulated genes, the most notable were members of the IL-17 receptor family (Il17rb, rc, and re), which were consistently downregulated during the entire feeding process while the pro-inflammatory cytokines IL-17a, c, d, and f were not expressed. Other downregulated genes were T-cell molecules CD28, GATA3, and retinoic acid-related orphan nuclear hormone receptor C (RORC), DNA repair molecule telomerase reverse transcriptase (TERT), basement membrane/ECM structural components HAPLN1 and LAMA1, neural adhesion molecule NCAM2, mitogenic pathway member JUN, WNT pathway members JUN and LEF1, hedgehog pathway members BMP2 and 4, and retinoic acid pathway member HOXA1.

### Modulation of gene expression during secondary infestations

**Figure 3:**
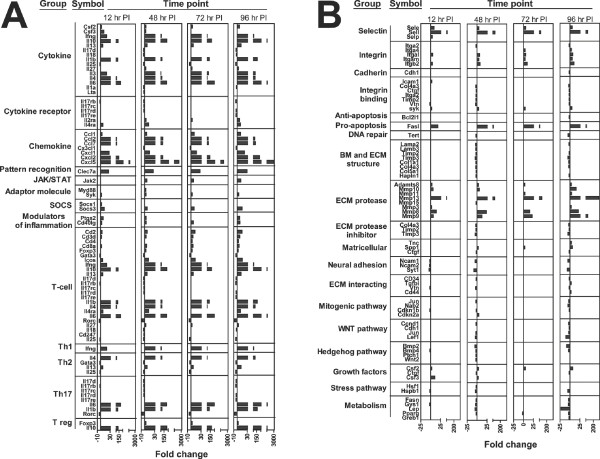
**Genes modulated during secondary infestations of mice compared to tick-free mice**. Gene expression during a secondary exposure of BALB/cJ mice to *I. scapularis *nymphs was measured using PCR arrays. Significant results were divided into groups as before. Official gene symbols and fold changes are shown at 12, 48, 72, and 96 hr post secondary infestation. The figure is split into sections A and B to aid viewing.

During the secondary infestation, Th1 and Th2 cytokines joined those upregulated on primary exposure. Interleukin-17 receptors remained downregulated, while IL-2ra and IL-4ra were upregulated. The expression profile of chemokines and PRR was similar to the primary infestation with the addition of CCL1. Cytokine signaling molecules JAK2, MYD88, SYK, SOCS1, and SOCS3 were upregulated. The CD40 ligand (CD40LG) joined (PTGS2) in the modulators of inflammation group. Many T-cell markers were upregulated along with Th1 and Th2 cytokines; however, transcriptional regulators important for CD4 T cell differentiation such as TBX21, GATA3, and RORC were unchanged or downregulated. The only exception was Forkhead box P3 (FOXP3), which was upregulated along with the cytokine IL-10, suggesting the possible involvement of T regulatory cells [20] (Figure 3A). All three selectins were upregulated, although SELP was only upregulated at 12 hr p.i. Integrins β-2, α-M, α-L, and α-4 were upregulated while α-2 was downregulated. Cadherins and integrin binding molecules were downregulated with the exception of SYK and ICAM1. Anti-apoptotic molecule BCL2L1 and DNA repair molecule TERT were downregulated while pro-apoptotic molecule FASL was upregulated. ECM proteases were strongly upregulated, but members of the BM/ECM structural molecule and ECM protease inhibitor groups were down regulated. With the exception of a few matricellular molecules, ECM interacting molecules, and growth factors, all the remaining groups were downregulated (Figure 3B).

### Array result validation

**Figure 4:**
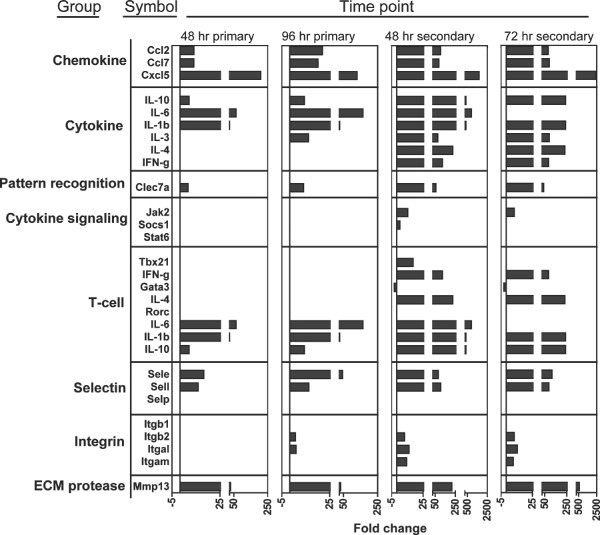
**Quantitative real-time PCR validation of PCR array data**. Based on the PCR array results, 25 genes were chosen for validation in a separate infestation experiment. Gene expression was measured at 48 and 96 hr p.i. during primary infestations and 48 and 72 hr p.i. during secondary exposures. All significant results are shown. In general, gene modulation supported the array study.

Based on the results of PCR array analysis as well as other studies reported in literature, twenty-five genes potentially involved in the host response to tick infestation were selected and further verified using quantitative real-time PCR (see additional file 1 for a gene list). Gene expression was determined at 48 and 96 hr p.i. for the primary infestation, and 48 and 72 hr p.i. for the secondary exposure. Twenty of the twenty-five genes tested showed a profile highly consistent with the PCR array results (Figure 4, additional file 4). In contrast, five genes (IL-3, GATA3, RORC, TBX21 and SELP) showed variable patterns of modulation. In particular, IL-3 upregulation was detected at 96 hours p.i. in the primary infestation. Downregulation of GATA3 was significant only in the secondary infestation while RORC downregulation was apparent at all time points but not significant. Lastly, TBX21 expression was upregulated at 48 hours p.i. in the secondary infestation and SELP up-regulation was not detected.

**Figure 5:**
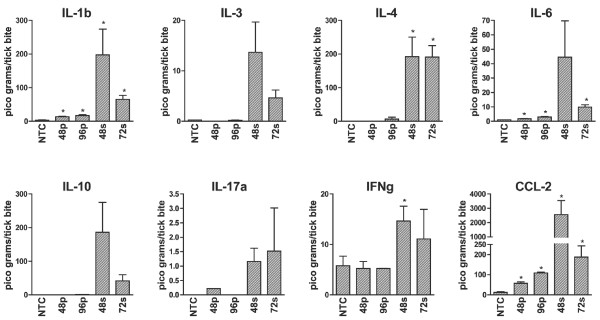
**Cytokine analysis of tick bite sites during primary and secondary infestations**. Concentrations of IL-1β, IL-3, IL-4, IL-6, IL-10, IL-17a, IFN-γ, and CCL2 were measured in skin biopsies from tick feeding sites at 48 and 96 hr p.i. primary infestation (48p and 96p) and 48 and 72 hr p.i. secondary infestation (48s and 72s) and compared to normal mouse skin using a two-tailed T-test; * indicates a p-value ≤ 0.05 compared to tick-free mice.

Regulation of protein expression may occur at many points between transcription and the production of functional protein. For this reason, the copies of an individual gene transcript may not reflect the expression of protein. To provide support for the transcriptome profile data we utilized an 8-analyte bioplex assay to measure protein expression by select cytokine and chemokine genes at the bite site (Figure 5). Analytes were chosen based on differentially modulated or biologically important molecules from the array data offered in bioplex format. Cytokines IL-1β, IL-4, IL-6, IFN-γ, and chemokine CCL-2 were significantly upregulated in agreement with the array and validation experiments. Interleukin-3, IL-10, and IL-17a showed similar but non-significant regulation. In order to directly compare protein and mRNA levels, fluorescent intensity values from the bioplex assay were converted to fold change over control sample fluorescence (Additional file 5). With the exception of low-abundance transcripts, these results suggest mRNA expression profiling at the tick-host interface could detect differences that correlate to the levels of expressed protein and can be a powerful tool for high-throughput functional analysis of the host cutaneous response to tick feeding.

## Discussion

### Primary infestation

During tick feeding, the cutaneous environment responds to skin injury by initiating innate defense mechanisms, shaping the ensuing adaptive immune response, and accommodating effector responses of adaptive immunity. In contrast, the feeding tick secretes an arsenal of salivary molecules that pharmacologically inhibit potentially unfavorable host responses [1]. The late initiation of host responses during primary infestation compared to secondary infestation is a striking example of tick-induced suppression of the host response. Early events at the bite site become measurable by 48 hours p.i. and include upregulation of CLEC7a, a lectin pattern recognition receptor. *I. scapularis *SALP15 has been shown to modulate dendritic cell (DC) function through the lectin receptor DC-SIGN [6]. Together these results suggest lectin pattern-recognition receptors may be important in initiation and modulation of anti-tick immunity. Ligation of CLEC7a induces the up-regulation of CXCL2 and IL-10 [21], molecules that were also upregulated in our study. Tick-induced expression of IL-10 has been previously reported and may represent a method of immune evasion by dampening pro-inflammatory responses [22,23].

Other cytokines upregulated early in the host response were IL-1β and IL-6. These are both potent pro-inflammatory cytokines suggesting a balance between anti-inflammatory IL-10 and pro-inflammatory IL-1β and IL-6 during the early host response to ticks. The presence of IL-1β and IL-6 at the bite site is supported by previous studies [24,25] and the concomitant upregulation of ICAM1, PTGS2 (COX2), CXCL1, CXCL2, CXCL5, and MMP13, molecules known to be induced by these cytokines [26-28]. The CXCL chemokines are potent chemoattractants for neutrophils, although they have also been shown to attract monocytes and mast cells [29]. CCL2 and CCL7 were originally described as macrophage chemotactic proteins (MCP) 1 and 3, reflecting their primary role as chemoattractants for macrophages, but they are also known to recruit basophils, eosinophils, NK cells, and DCs [29]. Recruitment of these cells into the bite site could be facilitated by the upregulation of SELL and ITGB2. These results suggest a model of immune activation during primary infestation where CLEC7a initiates neutrophil chemotaxis and anti-inflammatory cytokine production. Increased production of IL-1β and IL-6 by unknown mechanisms could play a role in promoting upregulation of chemokines specific for neutrophils and macrophages which in turn produce matrix metalloproteinases (MMP) and prostaglandins. Neutrophils are known to be present at the bite-site [30], but their role in anti-tick immunity is not well understood. Based on the previous identification of *I. scapularis *salivary proteins (ISL929 and 1373) that reduce super-oxide formation and expression of β-2 integrins in neutrophils treated with TNF-α [7], it is reasonable to assume they are important components of anti-tick immunity. These changes suggest reduced neutrophil ability to respond to tissue insult and destroy phagocytosed infectious agents.

Matrix metalloproteinases have a wide range of potential functions at the tick bite-site. MMP cleavage of ECM components exposes cryptic sites that have been associated with increased migration of leukocytes to the inflammatory focus; cleavage can also release bioactive molecules from the ECM. *I. scapularis *has been shown to possess a large family of salivary serine-protease inhibitors (serpins) that may be important in inhibiting host responses [31]. Immunization of rabbits with a serpin from *I. ricinus *resulted in increased tick mortality and reduced weight and fecundity in female ticks [32]. Since MMPs degrade and inactivate endogenous serpins [33], it is reasonable to hypothesize that MMPs contribute to host immunity by degrading tick-secreted serpins. MMPs also aid in angiogenesis and wound healing, processes that are inhibited by tick feeding [34]. Gene ontology gives general support to this analysis of the primary infestation. Significant terms from genes upregulated during primary infestation clustered into host response and biomineral formation groups. The host response category was dominated by chemokine, chemotaxis, cytokine, and immune response terms, although none of these terms were specific for any cell type. GO analysis also supported the role of upregulated genes as secreted molecules acting in the extracellular space. Analysis of downregulated genes during primary infestation identified nucleotide metabolism/transcription and SEFIR domain (IPR013568: SEFIR, an IL-17R domain) as significant. These terms are of interest for CD4 T cell differentiation (discussed below), and the possibility that tick feeding suppresses transcription during primary infestation. This is a potential mechanism behind the late induction of host responses during primary infestation.

### Secondary infestation

A second exposure to feeding by *I. scapularis *nymphs resulted in a faster and stronger host response as shown in Figures 3A and 3B. In contrast to the primary infestation, very significant gene modulation was evident by 12 hrs p.i. The genes modulated during primary infestations were also modulated during secondary infestations and were, in general, the genes with the highest fold-changes. Thus we postulate that genes upregulated during the primary infestation form a core host-response that drives anti-tick immunity even on repeated exposure.

### Migration

The migration of cells into an inflammatory focus is an important aspect of host immunity [29]. Resident cells must recognize skin damage by the feeding tick and secrete factors that enhance the recruitment of immune effectors to the bite site. Gene ontology analyses of upregulated genes during the secondary infestation strongly support the important role of chemotaxis in the anti-tick immune response. Specific GO terms suggested the migration of neutrophils, monocytes, other leukocytes, and lymphocytes into the bite site (Table 3). The upregulation of CCL1 was the only observed change in chemokine expression between primary and secondary infestation. Interestingly, this chemokine has been shown to attract Th2 and T regulatory cells [29]. Other upregulated genes known to support cell migration included selectins, integrins, and the integrin ligand ICAM1. While a number of alpha chain integrins were upregulated, the only beta chain upregulated was β-2. In support of previous reports that *I. scapularis *saliva inhibited endothelial cell expression of P selectin [35], our study showed only minimal upregulation of SELP that was not supported by later validation.

### Cytokines

Many additional cytokines were modulated during the secondary infestation when compared to the primary exposure (Figure 3A). These transcripts group together to form the cytokine cluster on gene ontology analysis, lending formal support to their importance in the anti-tick response. In particular, IL-4 and IL-13 were upregulated; these cytokines can be produced by Th2 cells, but also by basophils, eosinophils, and mast cells. Basophils have been shown to be indispensible for anti-tick immunity in models of infestation where acquired resistance occurs [36,37], and their migration into the bite site was supported by the upregulation of CCL chemokines and IL-3, which are chemotactic factors for basophils [38]. It is interesting to note the significant increase in expression of IL-4 and IL-13 during the secondary exposure despite the down regulation of IL-25, an important inducer of type-2 immunity [39]. In contrast to these type-2 cytokines, upregulation of IFN-γ and IL-27 could be due to the presence of T cells, Th1 cells, NK cells, and antigen presenting cells [40,41]. The upregulation of IFN-γ is surprising in light of previous reports of suppression by tick saliva [22], although negligible increases in expression have been previously reported in BALB/c mice infested with *I. scapularis *[23]. The mechanisms behind IFN-γ and IL-4 upregulation were strong enough to overcome the downregulation of IL-18, a known inducer of both cytokines [26]. Upregulation of colony stimulating factors 2 (GM-CSF or CSF2) and 3 (G-CSF or CSF3) and IL-3 suggests tick feeding may stimulate increased hematopoiesis and/or myelopoiesis. This possibility was supported by the gene ontology analysis (Table 3), previous reports of extramedullary erythropoiesis in tick-infested mice [42], and the downregulation of IL-17d, an inhibitor of hematopoietic progenitor colony formation [43]. Finally, our study also supports previously reported repression in the expression of tumor necrosis factor family members by tick salivary molecules [6,44]. In summary, the cytokine profile during secondary infestation presents a complex interplay between inducers and repressors of type 1 and type 2 immunity.

### T-cells

Th2 polarization of the cytokine response to tick feeding has been thoroughly documented by *in vitro *and *in vivo *studies [1]. For this reason, we sought to characterize the modulation of genes associated with T-cell and helper T-cell differentiation. During primary infestation, classic T-cell markers such as CD3, CD4, and CD8 did not significantly change, suggesting early T-cell involvement is minimal. Interestingly, the expression of co-stimulatory molecule CD28 was downregulated, which could be due to a lack of CD4 T-cell activation at the bite site, or the migration of CD28-expressing cells out of the skin. Genes related to Th17 differentiation, including the transcription factor RORC, IL-17, and the IL-17 receptors were either unchanged or downregulated, despite the high levels of IL-1β and IL-6. Most genes related to Th2 development were unchanged with the exception of GATA3, which was downregulated. GATA3 is an important transcription factor in Th2 development. Transcripts related to Th1 and T reg development were unchanged. These results suggest that during primary infestation of mice with *I. scapularis *nymphs, the cutaneous environment is not strongly polarized (or polarizing) toward any helper T-cell sub-set.

On secondary infestation, the upregulation of T-cell markers CD2, CD3, CD4, and CD8 suggested T-cell involvement at the bite-site. However, the polarization of CD4 T-cells remained equivocal. While Th2 cytokines IL-4 and IL-13 were upregulated, GATA3 remained repressed (Figure 3A). Similarly, the Th1 cytokine IFN-γ was upregulated, but IL-12 and TBX21 remained unresponsive. Th17-related transcripts were downregulated or unchanged. Interestingly, FOXP3 and IL-10 were upregulated, supporting a possible role for T regulatory cells at the bite site. In summary, results from the secondary exposure strongly suggests Th17 involvement at the bite-site is unlikely, while the remaining data shows a mixed Th1/Th2 cytokine profile and suggests the involvement of T regs. Failure to produce a polarized CD4 T cell response was also observed when keyhole limpet haemocyanin (KLH)-specific T cells were stimulated with KLH-loaded DCs in the presence of *Rhipicephalus sanguineus *tick saliva [8]. This implies that non-polarized CD4 T cell responses may be a common trait of anti-tick immunity and also supports our results at the protein/cellular level. Sialostatin L, an *I. scapularis *salivary protein, suppressed IL-17 production by lymph node cells during the induction of experimental autoimmune encephalomyelitis in mice [45]. In our results, significant Th17 suppression was observed (Figure 2, 3A) even from a *naïve *state, supporting the possibility that tick saliva contains potent suppressors of Th17 immunity.

### Signaling

Another focus of the present study was to uncover novel signaling pathways activated at the tick bite-site. Surprisingly, most genes related to the signaling pathways tested were either downregulated or unresponsive. Immunoreceptor signaling was a significant exception. Gene ontology results showed the largest gene cluster was related to immune cell signaling and activation. This is consistent with the rest of our results and suggests immunoreceptor signaling as a potential major pathway induced by tick feeding. However, we were unable to show any modulation of signal transducers and activators of transcription (STAT) or NFκB pathway molecules. The lack of STAT modulation in our study was surprising since STAT molecules are important effector molecules of cytokine signaling that induce their own expression [46]. Modulation or silencing of the NFκB pathway could be significant because of its vital role in the induction and regulation of immunity [47]. These results paired with the increase of SOCS transcripts suggest the tick bite site is characterized by both suppression and activation of immunoreceptor signaling.

Gene ontology analysis of the downregulated genes during secondary infestation showed only two significant terms: negative regulation of cell proliferation (GO_BP: 0008285) and SEFIR (IPR013568). This suggests the genes downregulated during secondary infestation do not fit into a common theme for GO enrichment. However, many groups and pathways were qualitatively downregulated. Transcripts related to integrin binding and neural adhesion were downregulated, indicating potential impairment of cell migration or adhesion-related signaling at the tick bite site. Increased cell turnover was suggested by the downregulation of genes encoding anti-apoptotic and DNA repair molecules. The downregulation of BM/ECM structure and ECM protease inhibitor groups combined with the upregulation of ECM proteases suggest significant modulation of ECM components. In addition to these groups, genes in classical pathways such as mitogenic, WNT, hedgehog, stress, and metabolism were downregulated. The WNT signaling pathway regulates a variety of cellular processes including cell proliferation, migration, and tissue morphogenesis. In canonical signaling, WNT stabilizes β-catenin that acts as a transcriptional co-activator by interacting with Lef/T-cell transcription factors (Tcfs) to regulate WNT target gene expression. Non-canonical signaling, on the other hand, is calcium-dependent and leads to activation of c-jun N-terminal kinase which plays a role in cell proliferation, differentiation, and apoptosis [48]. In addition to its role in developmental biology, the Hedgehog pathway has been shown to play a role in regulating regenerating cell populations [49]. Since cell proliferation, regeneration, and morphogenesis are involved in wound healing, epithelial maintenance, and hair follicle cycling, tick feeding may influence these processes. However, it is unclear whether this is a result of tick-saliva induced repression or a consequence of the inflammatory process at the bite-site lesion. In this regard, our infestation protocol avoided the use of capsules or any device to restrain the ticks during feeding that might have influenced the inflammatory reaction. In either case, our results qualitatively suggest the tick bite-site is characterized in part by the suppression of signaling molecule transcription.

## Conclusions

Our study supports a model of the tick-host interface where tick saliva inhibits gene transcription, Th17 immunity, and signal transduction molecule upregulation. In contrast, the host senses infestation through lectin PRRs and is primarily focused on the recruitment and subsequent activation of immune cells. During primary infestation, neutrophils and macrophages are recruited, while many additional cell types are recruited during secondary infestation. Host effector responses include a mixed Th1/Th2 CD4 T cell response, innate effector functions, a highly proteolytic environment, and increased cell turn-over. These responses are dampened by the action of T regulatory cells, SOCS, and IL-10.

To our knowledge, this is the first report of *in vivo *transcriptome profiling at the *I. scapularis *tick-host interface. Our results suggest tick feeding may activate favorable host responses such as the inhibition of gene transcription, downregulation of signaling molecules, and upregulation of inhibitors of inflammation while repressing unfavorable responses such as Th17 immunity. The mixed Th1/Th2 profile reported here is a novel finding that implies greater complexity to the host cutaneous response than previously reported. We believe this study will allow the rational design of further work probing *in vivo *mechanisms at the tick-host-pathogen interface.

## Competing interests

There are no competing interests in the publication of this work.

## Authors' contributions

DMH and FJA carried out the experimental work and drafted the manuscript. SKW acted as a consultant at all levels of the project. ST also acted as a consultant in the later stages of this project and provided significant support for all tick rearing activities. All authors read and approved the final manuscript.

## Supplementary Material

Additional file 1 **List of genes measured by qRT-PCR for validation of PCR array results**. Click here for file

Additional file 2 **Statistical comparison between primary and secondary infestations**. Significant changes in gene expression between primary and secondary infestation were measured using LIMMA and the same filtering criteria as before (Methods). Each column shows the genes significantly modulated over the primary infestation during a secondary exposure of mice to *I. scapularis *nymphs. Click here for file

Additional file 3 **Gene groups used to allow comparisons between time points**. All 233 genes measured in this study were grouped based on characteristics of the encoded protein. These groups were used to allow comparisons between time points throughout our study. Click here for file

Additional file 4 **Direct comparison of PCR array and qRT-PCR validation experiments**. Fold change and p-values obtained from the PCR array and qRT-PCR validation experiments are directly compared. "Primary" refers to primary infestation, while "secondary" refers to the secondary exposure. A "+" marks fold changes calculated from transcripts below the detection limit (Ct ≥ 34); red text denotes p-values ≤ 0.01. Click here for file

Additional file 5 **Comparison of PCR array, qRT-PCR validation, and protein expression levels**. Gene expression and protein levels were compared by transforming protein expression data into fold change in fluorescence intensity over control samples. "ns" refers to non-significant results (p-value > 0.01 for gene expression and > 0.05 for protein expression); "+" denotes fold changes calculated from transcripts below the detection limit (Ct ≥ 34); "nt" refers to genes not tested. Click here for file
