# Supplementary material for: Transcriptional profiling of the murine cutaneous response during initial and subsequent infestations with Ixodes scapularis nymphs
Source: Parasit Vectors. 2012 Feb 6;5:26. doi: 10.1186/1756-3305-5-26 (PMC3293053; doi:10.1186/1756-3305-5-26)
Supplement: Additional file 1 — List of genes measured by qRT-PCR for validation of PCR array results. [file 1756-3305-5-26-S1.PDF]

List of genes measured by qRT-PCR for validation of PCR array results.

Gene symbol	Gene Name
Ccl2	Chemokine (C-C motif) ligand 2
Ccl7	Chemokine (C-C motif) ligand 7
Ccr5	Chemokine (C-C motif) ligand 5
Clec7a	C-type lectin domain family 7, member a
Cxcl5	Chemokine (C-X-C motif) ligand 5
Gata3	GATA binding protein 3
IFN-γ	Interferon gamma
IL-10	Interleukin 10
IL-1β	Interleukin 1 beta
IL-3	Interleukin 3
IL-4	Interleukin 4
IL-6	Interleukin 6
Itgal	Integrin alpha L
Itgam	Integrin alpha M
Itgb1	Integrin beta 1 (fibronectin receptor beta)
Itgb2	Integrin beta 2
Jak2	Janus kinase 2
Mmp13	Matrix metallopeptidase 13
Rorc	RAR-related orphan receptor gamma
Sele	Selectin, endothelial cell
Sell	Selectin, lymphocyte
Selp	Selectin, platelet
Socs1	Suppressor of cytokine signaling 1
Stat6	Signal transducer and activator of transcription 6
Tbx21	T-box 21
Actb	Actin, beta
Gadph	Glyceraldehyde-3-phosphate dehydrogenase
Gusb	Glucuronidase, beta
Hprt1	Hypoxanthine guanine phosphoribosyl transferase 1
Hsp90ab1	Heat shock protein 90 alpha (cytosolic), class B member 1
