# Supplementary figures and images for: Transcriptional profiling of the murine cutaneous response during initial and subsequent infestations with Ixodes scapularis nymphs
Source: Parasit Vectors. 2012 Feb 6;5:26. doi: 10.1186/1756-3305-5-26 (PMC3293053; doi:10.1186/1756-3305-5-26)

**Statistical comparison between primary and secondary infestations**

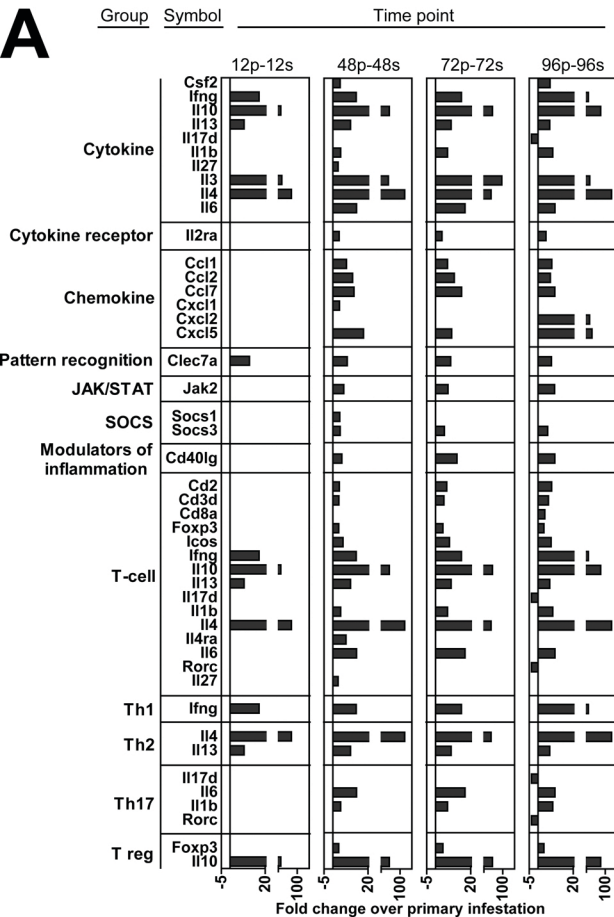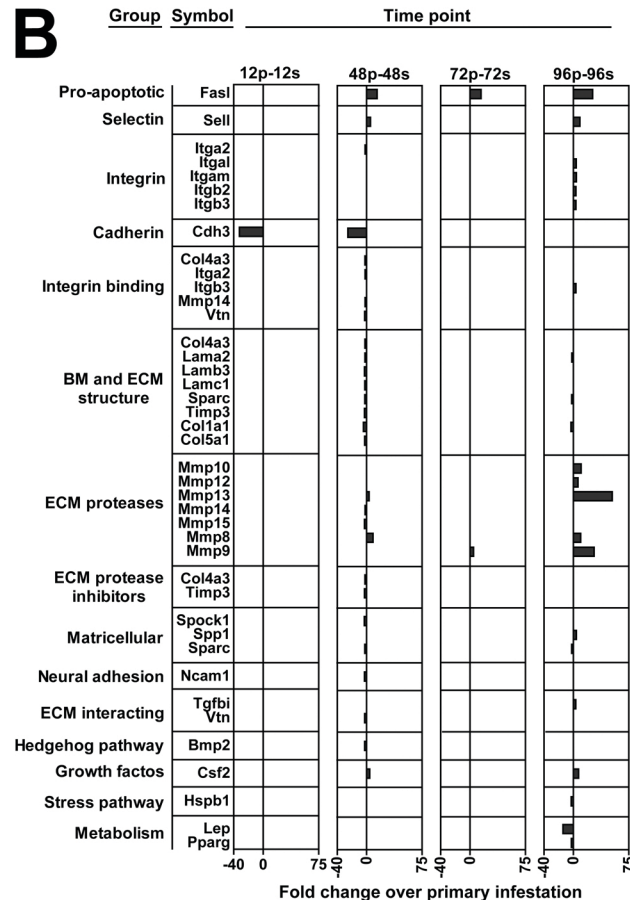

Supplement: Additional file 2 — Statistical comparison between primary and secondary infestations. Significant changes in gene expression between primary and secondary infestation were measured using LIMMA and the same filtering criteria as before (Methods). Each column shows the genes significantly modulated over the primary infestation during a secondary exposure of mice to I. scapularis nymphs. [file 1756-3305-5-26-S2.PDF]
