# Supplementary material for: Transcriptional profiling of the murine cutaneous response during initial and subsequent infestations with Ixodes scapularis nymphs
Source: Parasit Vectors. 2012 Feb 6;5:26. doi: 10.1186/1756-3305-5-26 (PMC3293053; doi:10.1186/1756-3305-5-26)
Supplement: Additional file 4 — Direct comparison of PCR array and qRT-PCR validation experiments. Fold change and p-values obtained from the PCR array and qRT-PCR validation experiments are directly compared. "Primary" refers to primary infestation, while "secondary" refers to the secondary exposure. A "+" marks fold changes calculated from transcripts below the detection limit (Ct ≥ 34); red text denotes p-values ≤ 0.01. [file 1756-3305-5-26-S4.PDF]

Direct comparison of PCR array and qRT-PCR validation experiments.

	48 hr primary array		48 hr primary validation		96 hr primary array		96 hr primary validation	
gene	fold change	adj. p value	fold change	adj. p value	fold change	adj. p value	fold change	adj. p value
Ccl2	8.85	2.68E-03	8.7	2.43E-10	12.1	4.52E-04	20.5	2.36E-12
Ccl7	7.94	5.06E-04	8.66	6.02E-12	11	1.59E-04	17.7	3.37E-13
Clec7a	3.14	1.47E-03	5.09	5.44E-05	3.9	3.90E-02	8.61	1.95E-05
Cxcl5	121	3.29E-04	208	4.62E-15	68.5	2.43E-03	130	4.21E-11
Gata3	0.372	1.47E-03	0.661	2.21E-02	0.422	9.97E-03	0.716	5.68E-02
IFNg	3.79^{+}	1.71E-02	1.94^{+}	1.94E-01	1.5^{+}	4.50E-01	6.62^{+}	1.62E-03
IL1b	16.7	1.62E-04	25.3	8.11E-12	20.4	1.45E-03	27.4	1.81E-09
IL10	4.51	1.46E-03	5.71	4.15E-10	5.19	2.74E-02	9.17	1.19E-08
IL3	0.588^{+}	3.34E-01	2.8^{+}	3.08E-02	2.75^{+}	2.68E-01	11.7	1.21E-05
IL4	0.597	9.10E-02	1.04^{+}	9.58E-01	0.738	4.58E-01	3.85^{+}	1.80E-01
IL6	17.5	4.58E-04	64.1	1.84E-08	51.1	1.33E-03	165	8.00E-09
Itgal	2.58	1.02E-02	2.46	1.75E-02	1.9	5.54E-02	4	2.56E-03
Itgam	2.7	1.92E-04	2.51	3.22E-07	1.51	1.58E-01	2.68	7.64E-06
Itgb1	1.1	3.49E-01	0.801	6.50E-02	1.02	9.03E-01	1.03	8.53E-01
Itgb2	3.04	1.92E-04	2.66	2.34E-03	2.89	5.85E-03	3.57	1.18E-03
Jak2	1.11	5.38E-01	1.32	1.36E-01	1.17	4.95E-01	2.25	4.12E-04
Mmp13	20.3	6.32E-06	30.1	9.62E-07	6.26	1.43E-03	33	1.21E-05
Rorc	0.249	5.87E-04	0.666	6.32E-01	0.501	9.97E-03	1.12	8.97E-01
Sele	1.87	1.33E-03	14.9	8.01E-03	2.66	3.45E-03	43.8	1.55E-03
Sell	14.9	1.92E-04	11.4	1.58E-05	7.87	2.70E-03	12	5.46E-05
Selp	1.9	2.91E-03	1.51	6.97E-05	1.56	5.13E-02	2.08	1.21E-05
Socs1	0.719	1.90E-01	0.679	4.11E-02	1.31	1.92E-01	1.16	4.77E-01
Stat6	0.826	3.31E-01	0.875	1.46E-01	0.911	6.04E-01	0.983	8.69E-01
Tbx21	0.753^{+}	3.06E-01	1.36^{+}	6.26E-01	0.963^{+}	9.01E-01	3.18^{+}	6.13E-02

	48 hr secondary array		48 hr secondary validation		72 hr secondary array		72 hr secondary validation	
gene	fold change	adj. p value	fold change	adj. p value	fold change	adj. p value	fold change	adj. p value
Ccl2	99.7	1.68E-05	103	6.37E-18	67.2	6.00E-05	81.6	3.32E-15
Ccl7	95.8	2.08E-06	88.1	4.47E-19	76.6	2.32E-06	90.6	2.06E-15
Clec7a	25.8	5.32E-06	56.3	9.84E-12	27.3	2.32E-06	39	5.04E-09
Cxcl5	2120	1.77E-06	1680	1.16E-18	1460	1.07E-06	2390	1.14E-17
Gata3	0.323	1.48E-04	0.44	5.64E-06	0.333	2.29E-03	0.436	2.34E-04
IFNg	50.7	5.90E-05	121	2.53E-10	33.7	5.85E-05	84.9	1.17E-08
IL1b	74.3	5.32E-06	102	2.26E-15	114	1.45E-06	107	1.32E-13
IL10	248	1.77E-06	343	7.73E-21	191	4.00E-06	239	4.16E-17
IL3	30.1	3.71E-04	74.8	2.83E-11	78	2.37E-04	90.9	2.05E-10
IL4	71.6	5.70E-05	225	3.98E-07	33.1	1.13E-04	234	9.30E-06
IL6	237	5.86E-06	903	5.93E-13	292	4.00E-06	2.8^{+}	3.64E-01
Itgal	6.58	4.04E-04	11.7	2.71E-07	5.99	5.74E-03	10.5	1.13E-05
Itgam	4.91	1.52E-04	9.43	6.93E-15	4.29	7.90E-03	6.68	8.08E-11
Itgb1	1.01	9.60E-01	1.16	1.88E-01	1.18	4.59E-01	1.21	1.83E-01
Itgb2	7.83	1.37E-05	7.51	3.52E-07	9.68	1.60E-04	7.77	1.09E-05
Jak2	7.02	6.07E-05	10.4	4.01E-11	7.7	8.51E-05	7.69	7.96E-09
Mmp13	69.5	1.37E-05	215	1.69E-10	85.3	1.13E-04	582	5.70E-10
Rorc	0.137	8.06E-05	0.429	2.80E-01	0.196	2.29E-03	0.565	5.46E-01
Sele	4.77	6.94E-04	81.9	4.53E-05	5.92	1.60E-04	116	3.03E-04
Sell	75.5	7.04E-07	105	9.94E-11	59.2	5.29E-05	88.8	5.51E-09
Selp	2.43	5.83E-04	2.41	7.71E-10	1.9	4.97E-02	1.78	1.09E-05
Socs1	2.87	6.06E-04	3.04	2.75E-07	2.29	1.51E-02	1.6	5.42E-02
Stat6	0.856	3.49E-01	0.797	2.93E-02	1.01	9.82E-01	0.563	5.60E-02
Tbx21	2.35^{+}	5.19E-02	15.6	1.15E-05	1.74^{+}	1.07E-01	3.82^{+}	9.16E-02

"+" marks values below detection limit (Ct > 34)
Red text indicates p value ≤ 0.01
