# Supplementary material for: Transcriptional profiling of the murine cutaneous response during initial and subsequent infestations with Ixodes scapularis nymphs
Source: Parasit Vectors. 2012 Feb 6;5:26. doi: 10.1186/1756-3305-5-26 (PMC3293053; doi:10.1186/1756-3305-5-26)

Comparison of array, qRT-PCR, and protein levels. Protein levels are expressed as fold change in fluorescence over control samples. ns=not significant (p>0.01 for array or qRT-PCR or p>0.05 for protein); "+" indicates transcripts below detection limit (Ct>34); nt=not tested.

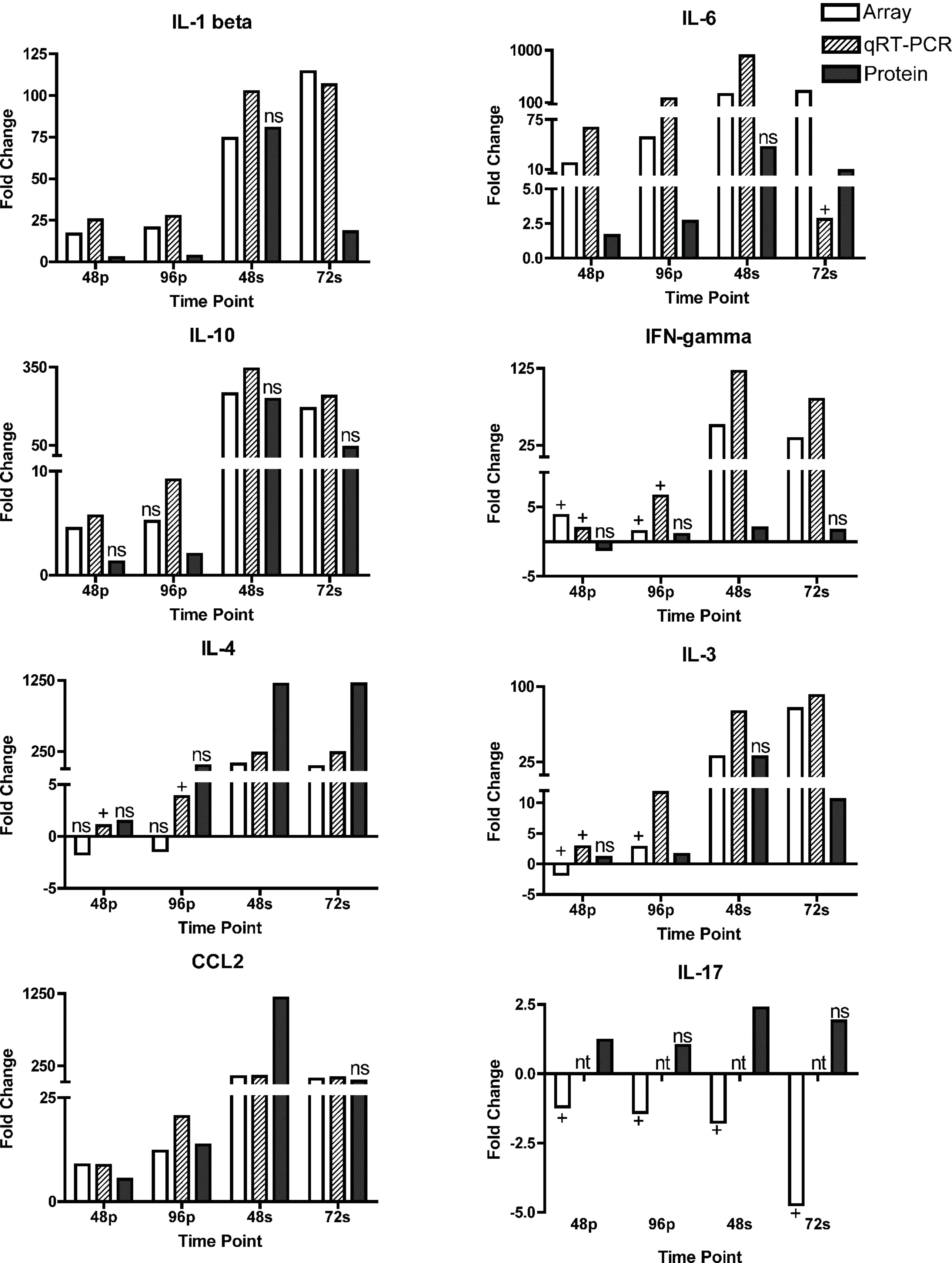

Supplement: Additional file 5 — Comparison of PCR array, qRT-PCR validation, and protein expression levels. Gene expression and protein levels were compared by transforming protein expression data into fold change in fluorescence intensity over control samples. "ns" refers to non-significant results (p-value > 0.01 for gene expression and > 0.05 for protein expression); "+" denotes fold changes calculated from transcripts below the detection limit (Ct ≥ 34); "nt" refers to genes not tested. [file 1756-3305-5-26-S5.PDF]
